# Survival risk stratification based on prognosis nomogram to identify patients with esophageal squamous cell carcinoma who may benefit from postoperative adjuvant therapy

**DOI:** 10.1186/s12885-024-13085-w

**Published:** 2024-10-29

**Authors:** Yumeng Zhang, Weilin Xu, Mengxing Wu, Yurong Li, Guanhua Chen, Yu Cheng, Xinchen Sun, Liang Yang, Shu Zhou

**Affiliations:** 1 Department of Radiation Oncology, Shanghai First Maternity and Infant Hospital, School of Medicine, Tongji University, Shanghai, 200092 China; 2 Department of Radiation Oncology, The First Affiliated Hospital of Nanjing Medical University, Nanjing, 210029 China; 3 Department of Radiation Oncology, The Affiliated Wuxi People’s Hospital of Nanjing Medical University, Wuxi People’s Hospital, Wuxi, 214023 Jiangsu China; 4 The Second Affiliated Hospital, Zhejiang University School of Medicine, Hangzhou, 310009 China; 5 Department of Radiation Oncology, Nanjing Jinling Hospital, Affiliated Hospital of Medical School, Nanjing University, Nanjing, 210029 China; 6 Department of Oncology, The Second Hospital of Nanjing, Nanjing University of Chinese Medicine, Nanjing, 210029 China

**Keywords:** Esophageal squamous cell carcinoma, Adjuvant therapy, Prognosis, Nomogram, Risk stratification

## Abstract

**Objective:**

The purpose of the study is to develop a prognosis nomogram for esophageal squamous cell carcinoma (ESCC) patients with radical resection and to identify patients who may benefit from postoperative adjuvant radiotherapy/chemoradiotherapy through survival risk stratification.

**Methods:**

We retrospectively enrolled patients who underwent esophagectomy in the First Affiliated Hospital of Nanjing Medical University from July 2015 to June 2017. Patients with stage I-III esophageal squamous cell carcinoma who received radical R0 resection with or without postoperative adjuvant radiotherapy/chemoradiotherapy were included. Further, patients were randomly allocated into two groups (training and validation cohorts) with a distribution ratio of 7:3. The prognosis nomogram was constructed based on independent factors determined by univariate and multivariate Cox analyses. The area under the receiver operating characteristic curve (AUC) and calibration curve were adopted to evaluate the discriminative ability and reliability of the nomogram. The accuracy and clinical practicability were respectively assessed by C-index values and decision curve analysis (DCA), and further contrasted the nomogram model and the eighth edition of the American Joint Committee on Cancer (AJCC) TNM staging system. In addition, survival risk stratification was further performed according to the nomogram, and the effect of postoperative adjuvant therapy on each risk group was appraised by the Kaplan-Meier survival analysis.

**Results:**

A total of 399 patients with esophageal squamous cell carcinoma were recruited in this study, including the training cohort (*n* = 280) and the validation cohort (*n* = 119). The nomogram-related AUC values ​​for 1, 3, and 5-year OS were 0.900, 0.795, and 0.802, respectively, and 0.800, 0.865, 0.829 in the validation cohort, respectively. The slope of the calibration curve for both cohorts was close to 1, indicating good consistency. The C-index value of the nomogram was 0.769, which was higher than that of the AJCC 8th TNM staging system by 0.061 (*p* < 0.001). Based on the prognosis nomogram, patients were stratified into three risk groups (low, medium, and high), and there were obvious differences in prognosis among the groups (*p* < 0.001). Furthermore, postoperative adjuvant therapy has been shown to enhance the 5-year survival rate by over 15% among patients classified as medium- and high-risk.

**Conclusion:**

The constructed nomogram as developed resulted in accurate and effective prediction performance in survival outcomes for patients with stage I-III esophageal squamous cell carcinoma who underwent radical R0 resection, which is superior to the AJCC 8th TNM staging system. The survival risk stratification had potential clinical application to guide further personalized adjuvant therapy.

**Supplementary Information:**

The online version contains supplementary material available at 10.1186/s12885-024-13085-w.

## Introduction

Esophageal cancer stands as a prevalent malignancy of the digestive tract on a global scale. According to the International Agency for Research on Cancer (https://gco.iarc.fr), Esophageal cancer ranks seventh in incidence and sixth in mortality among all cancers globally [1], with significant geographic variation in epidemiology. The highest incidence rates are found in East Asia, particularly in China, where more than 50% of cases occur [2]. Pathologically, esophageal cancer is predominantly composed of two main types: Esophageal Squamous Cell Carcinoma (ESCC) and Esophageal Adenocarcinoma (EAC), each exhibiting distinct histological behaviors, geographic features, and etiologies. Although the prevalence of EAC has observed a substantial rise in recent years, ESCC still accounts for approximately 90% of all esophageal cancer cases, mainly occurring in Asia, East Africa, and South America [3]. ESCC can be influenced by various risk factors, encompassing alcohol consumption, smoking, dietary patterns, malnutrition, genetic predisposition, etc [4]. , but the etiology is not yet fully elucidated. In addition, esophageal cancer is insidious, highly invasive, and has a high tumor mutation burden, with a five-year overall survival rate standing at a mere 20% [5, 6].

At present, surgery remains the primary treatment for resectable ESCC. Nevertheless, there is a potential for local recurrence and distant metastasis even after undergoing surgical intervention. It is worth noting that the five-year survival rate for patients who undergo simple surgery is still below 60% [7–9]. Despite surgery combined with neoadjuvant chemotherapy and radiotherapy being recognized as the standard treatment for esophageal cancer, a significant majority of patients with this condition still opt for surgical resection as their primary treatment. Nowadays, a unanimous agreement regarding the appropriate postoperative adjuvant therapy for esophageal cancer is lacking in scholarly discourse. The primary issue lies in the absence of a definitive identification of the specific population that derives advantages from such therapy. According to the clinical practice guidelines for esophageal cancer from the National Comprehensive Cancer Network (NCCN), patients who have undergone R0 resection for ESCC are not advised to receive any adjuvant therapy [10]. However, several retrospective studies in China have suggested that adjuvant radiotherapy/chemoradiotherapy after surgery can improve the disease-free survival and overall survival (OS) of IIB-III stage patients who have received radical surgery for esophageal squamous cell carcinoma [11–13]. This conclusion was further confirmed in a prospective randomized III clinical trial reported by the team of Xiao Zefen [14, 15]. As evidenced by prior research findings, the survival rates of ESCC patients with T3 stage and N + stage can be enhanced through the implementation of postoperative adjuvant therapy [16–22]. A propensity-matched analysis study also reported that postoperative adjuvant radiotherapy/chemotherapy can not only benefit patients with T3-4 stage and N+, but also improve the survival of patients with larger tumors, low differentiation, and R1/2 resection [23]. Therefore, we believe that constructing an individualized prediction model utilizing the clinical and pathological characteristics associated with the prognosis of ESCC patients in conjunction with the American Joint Committee on Cancer (AJCC) 8th TNM staging may yield more precise identification of the groups benefiting from postoperative adjuvant therapy.

Traditionally, the AJCC TNM staging system has been broadly regarded as a clinical tool for assessing and forecasting the course of esophageal cancer. It provides crucial information about tumor invasion depth, regional lymph node involvement, and the presence of distant metastasis, aiding in treatment decisions and prognostic evaluations. However, the AJCC 8th TNM staging system falls short in completely individualizing the prediction of the survival rate of ESCC patients who undergo radical surgery, so it is essential to thoroughly consider other tumor characteristics and demographic factors that have a significant impact on survival outcomes [24, 25]. As a visual statistical tool, the nomogram can accurately predict individualized risks, which is convenient for clinicians and patients to evaluate the survival outcomes of the disease. Given the limitations of the AJCC staging system, the nomogram integrating important prognostic variables (such as age, sex, tumor length, differentiation degree, etc.) has shown higher clinical utility in predicting risk [26].

The primary objective of this study is to construct and validate a nomogram prediction model for patients with stage I-III ESCC who have undergone R0 surgery, with or without adjuvant radiotherapy /chemoradiotherapy. Additionally, the study aims to establish a risk stratification and prognostic scoring system for the entire patient population based on the constructed prediction model to further explore the impact of postoperative adjuvant therapy on patient survival in each risk group. This will facilitate the identification of patients who are most likely to benefit from postoperative adjuvant therapy for esophageal squamous cell carcinoma (ESCC), thereby enabling more targeted and individualized clinical treatment decisions.

## Materials and methods

### Patient selection

This retrospective study analyzed patients who underwent radical surgery for ESCC at the First Affiliated Hospital of Nanjing Medical University between July 2015 and June 2017. The inclusion criteria were as follows: (1) the patients with thoracic ESCC; (2) ESCC as the primary tumor; (3) radical esophagectomy as initial treatment; (4) with or without postoperative adjuvant therapy (radiotherapy/chemoradiotherapy). Patients were excluded if they met any of the following criteria: (1) non-ESCC (such as adenocarcinoma, neuroendocrine carcinoma, adenosquamous carcinoma, mixed carcinoma, or undifferentiated carcinoma) as the primary tumor; (2) with neoadjuvant therapy before operation; (3) with postoperative chemotherapy alone; (4) R1 or R2 surgery; (5) unclear T stage; (6) IV stage; (7) death within 3 months after esophagectomy; (8) lost follow-up within 6 months after surgery.

### Data collection

The personal information, clinical and pathological characteristics, the process of the treatment, and imaging examinations were collected through the hospital information system. Specific information is as follows: age, sex, body mass index (BMI), performance status (PS), having basic diseases, smoking habit, alcohol consumption habit, family history, tumor location, tumor length, differentiation grade, lymphovascular space invasion (LVSI), Perineuronal invasion, lymph node ratio (LNR), surgical approach, pathological T stage, pathological N stage, pathological TNM stage, and specific postoperative treatment. BMI was classified into four levels according to the World Health Organization standard; PS was scored using the Eastern Cooperative Oncology Group (ECOG) five-point scale; tumor staging was determined using the 8th edition of the AJCC TNM pathological staging system; tumor location was considered the location of the tumor center; and LNR was defined as the ratio of the number of positive lymph nodes to the total number of lymph nodes removed during esophagectomy.

### Diagnosis and treatment

It is necessary for patients to undergo a comprehensive evaluation before radical surgery. Esophagoscopy and pathological biopsy are used for diagnosis, and all patients are confirmed by pathology before surgery. All Patients are required to receive physical examinations, blood routine, urine routine, stool routine, coagulation function, serum tumor markers, liver function, renal function, heart function, and lung function examination. In order to confirm the stage of cancer, more examinations need to be performed, including endoscopic ultrasonography of the esophagus, double contrast radiography of the upper gastrointestinal, esophageal magnetic resonance imaging (MRI), enhanced computed tomography (CT) of the neck, chest, and abdomen, abdominal ultrasound, radionuclide whole body bone scan, brain MRI and positron emission tomography (PET)-CT.

In the study, a minority of patients underwent endoscopic surgery completely (*n* = 7), while the majority were treated with traditional open operation for esophageal cancer. Therefore, surgical approaches were recorded as Sweet esophagectomy, Ivor Lewis esophagectomy, and McKeown esophagectomy, which are chosen based on the specific location and stage of the tumor. The surgery requires achieving radical resection and dissection of regional lymph nodes in the mediastinum and upper abdomen.

The commencement of postoperative radiotherapy or postoperative chemoradiotherapy for patients who received adjuvant treatment in this study took place within a timeframe of 4–8 weeks following the surgical procedure. The multi-disciplinary team guided specific adjuvant treatment plans. Intensity-modulated radiation therapy (IMRT) is the key technology for postoperative adjuvant radiotherapy in our center. The total prescribed dose was 45–50.4 Gy, with a conventional fractionation pattern (1.8–2.0 Gy/fraction, 5 fractions/week). Postoperative adjuvant radiotherapy and chemotherapy included sequential and concurrent chemoradiotherapy. Therefore, the chemotherapy regimens used in postoperative adjuvant chemoradiotherapy were not the same in this study. The concurrent chemotherapy regimens mainly included: (1) S-1 (tegafur, gimeracil, and oteracil potassium) monotherapy; (2) paclitaxel combined with cisplatin; (3) docetaxel combined with cisplatin; (4) fluorouracil combined with cisplatin. Except for S-1, all chemotherapy regimens had a 21-day cycle. Two cycles of concurrent chemotherapy are performed during postoperative radiotherapy and/or 4–6 cycles of sequential chemotherapy are followed by radiotherapy.

### Study endpoints and follow-up

The primary objective of this investigation was to evaluate the overall survival (OS), which was defined as the interval from the date of the surgical procedure to the date of death from any cause or the date of the last follow-up. A systematic follow-up schedule was implemented, commencing one month after surgery and continuing at three-month intervals for the initial two years postoperatively. Subsequently, follow-ups occurred every six months for the third to fifth year, and annually thereafter. The last follow-up was performed in October 2021. During the follow-up visits, physical examination, routine laboratory tests (such as blood routine, liver and kidney function, tumor markers, etc.), and necessary imaging examinations (such as neck/chest/abdominal CT, esophagography, esophagogastroduodenoscopy, cervical/abdominal lymph node ultrasound, etc.) were performed. Follow-up was conducted through both regular outpatient visits and telephone interviews. The survey instrument designed specifically for this study, pertaining to the follow-up, is comprehensively described in the **Supplementary File**.

### Statistical methods

In this study, X-Tile 3.6.1 software was utilized to calculate the cutoff values of continuous variables (such as age, tumor length, and LNR) in baseline data, and then describe them as binary variables [27]. At a ratio of 7:3, all patients were randomly categorized into a training cohort and a validation cohort. In the training cohort, screened variables with *p* < 0.05 by univariate Cox analysis were included in the multivariate Cox regression analysis. Subsequently, a nomogram predicting the 1-, 3-, and 5-year survival rates of postoperative ESCC patients was developed based on statistically significant prognostic factors (*p* < 0.05). The validation of the nomogram consists of two portions, including the internal validation implemented by the bootstrap method with 1000 resamples and the external validation based on the validation cohort. The discriminative ability and consistency of the nomogram prediction model were validated by the area under the receiver operating characteristic (ROC) curve (AUC) and calibration curve. Furthermore, decision curve analysis (DCA) was employed to compare the AJCC 8th staging system with the nomogram prediction model. The evaluation of the clinical effectiveness of models is frequently conducted using the Decision Curve Analysis (DCA), which assesses the net benefits across a range of threshold probabilities. This evaluation method incorporates various considerations, including patient and physician preferences, in order to provide a comprehensive understanding of the model’s utility [28]. Additionally, the X-Tile 3.6.1 software was also used to calculate the cutoff value of the total score of patients in the nomogram model, dividing all patients into low-risk, medium-risk, and high-risk groups. Kaplan-Meier method was used to analyze the impact of adjuvant therapy on survival in each risk group [27].

The statistical significance of all tests conducted in this study was determined using a two-sided approach, with a threshold of *p* < 0.05. The data analysis was performed using R 4.0.5 (http://www.r-project.org), GraphPad Prism 9.0 (GraphPad, CA, USA), and SPSS 26.0 (IBM Corp: Armonk, NY).

## Result

### Clinical characteristics and survival of patients

**Fig. 1:**
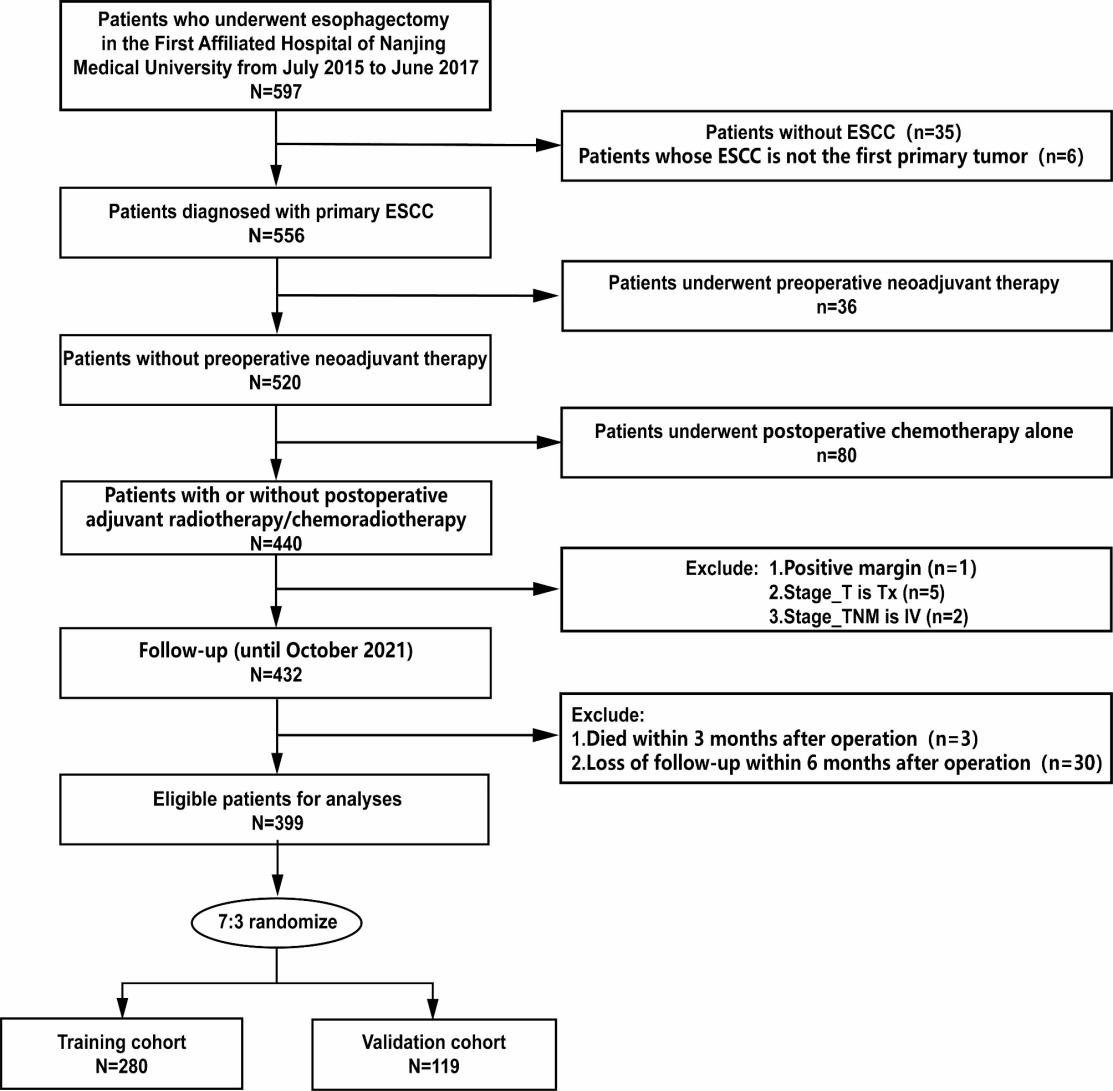
Flow chart of patient selection

A total of 399 patients who underwent curative surgery for ESCC at the First Affiliated Hospital of Nanjing Medical University were included in this study (Fig. 1), including 280 (70%) patients in the training cohort, and 119 (30%) patients in the validation cohort. The clinical and pathological attributes of the individuals are concisely outlined in Table 1 (and Table S1 in supplementary material). In this study, there were 270 patients who underwent curative surgery alone and 129 patients who received sequential postoperative adjuvant therapy (including 26 patients who received postoperative radiotherapy and 103 patients who received postoperative chemoradiotherapy). In the overall population, most patients (350/399) were under the age of 71 years. The male patient population outnumbered the female patient population by approximately threefold (74.2% vs. 25.8%). Approximately half of the patients had a smoking history, and one-third had an alcohol history. The distribution of these clinical characteristics was similar between the group receiving surgery alone and the group receiving postoperative adjuvant therapy, as well as in the overall population. Tumors were mostly located in the middle and lower thoracic segments. In the group receiving postoperative adjuvant therapy, the occurrence of tumors measuring 3 cm or larger was more than that of the surgery-alone group (68.2% vs. 44.4%). In terms of histological grade, more than 90% were moderately or poorly differentiated. The incidence of LVSI was 23.3% in the postoperative adjuvant therapy group compared to 8.9% in the surgery alone group, with a similar trend observed for nerve invasion (18.6% vs. 9.3%). Less than 10% of patients in the surgery alone group had an LNR ≥ 12% (90.7% vs. 9.3%), while nearly 30% of patients in the postoperative adjuvant therapy group had an LNR ≥ 12% (72.1% vs. 27.9%). In both study groups, the Ivor-Lewis esophagectomy was the predominant surgical approach, representing approximately 75% of all procedures performed. In terms of postoperative pathological staging, stage I-III patients were evenly distributed in the overall population, with each stage accounting for almost one-third of the population. In the surgery alone group, stage I patients accounted for 43.0%, while in the postoperative adjuvant therapy group, stage III patients accounted for 67.4%.

As of October 2021, the median follow-up time for the overall population was 62 months (5–74 months). The 1-year, 3-year, and 5-year survival rates for the overall ESCC patients were 93.2%, 79.6%, and 71.7%, respectively. Among those who received surgery alone, the survival rates at 1 year, 3 years, and 5 years were 93.3%, 83.6%, and 76.3%, respectively. In contrast, the patients who underwent postoperative adjuvant therapy had survival rates of 93.0%, 71.3%, and 62.1% at the same time intervals. This study included a considerable proportion of patients with stage I esophageal squamous cell carcinoma, accounting for almost one-third of the total. It is worth noting that patients with stage I who underwent surgery exhibited a relatively high survival rate. However, neither group of patients achieved the median survival time.

**Table 1:** Baseline clinicopathologic characteristics of selected patients

Characteristics	The entire cohort			The training cohort	The validation cohort
	All patients	Surgery	Surgery + Adjuvant	*N* = 280(%)	*N* = 119(%)
	*N* = 399(%)	*n* = 270(%)	*n* = 129(%)		
Age					
≤71	350(87.7)	232(85.9)	118(91.5)	247(88.2)	103(86.6)
> 71	49(12.3)	38(14.1)	11(8.5)	33(11.8)	16(13.4)
Sex					
Male	296(74.2)	191(70.7)	105(81.4)	213(76.1)	83(69.7)
Female	103(25.8)	79(29.3)	24(18.6)	67(23.9)	36(30.3)
BMI					
<18.5	29(7.3)	21(7.8)	8(6.2)	20(7.1)	9(7.6)
18.5–24.9	283(70.9)	190(70.4)	93(72.1)	204(72.9)	79(66.4)
25-29.9	82(20.6)	56(20.7)	26(20.2)	53(18.9)	29(24.4)
≥30	5(1.3)	3(1.1)	2(1.6)	3(1.1)	2(1.7)
Having basic diseases					
No	227(56.9)	152(56.3)	75(58.1)	158(56.4)	69(58.0)
Yes	172(43.1)	118(43.7)	54(41.9)	122(43.6)	50(42.0)
Smoking habit					
No	218(54.6)	153(56.7)	65(50.4)	141(50.4)	77(64.7)
Yes	181(45.4)	117(43.3)	64(49.6)	139(49.6)	42(35.3)
Alcohol consumption habit					
No	272(68.2)	194(71.9)	78(60.5)	183(65.4)	89(74.8)
Yes	127(31.8)	76(28.1)	51(39.5)	97(34.6)	30(25.2)
Family history					
No	353(88.5)	233(86.3)	120(93.0)	248(88.6)	105(88.2)
Yes	46(11.5)	37(13.7)	9(7.0)	32(11.4)	14(11.8)
EOCG					
0	117(29.3)	105(38.9)	12(9.3)	84(30.0)	33(27.7)
1	244(61.2)	142(52.6)	102(79.1)	172(61.4)	72(60.5)
2	38(9.5)	23(8.5)	15(11.6)	24(8.6)	14(11.8)
Tumor location					
Upper	20(5.0)	12(4.4)	8(6.2)	16(5.7)	4(3.4)
Middle	176(44.1)	117(43.3)	59(45.7)	118(42.1)	58(48.7)
Lower	203(50.9)	141(52.2)	62(48.1)	146(52.1)	57(47.9)
Tumor length(cm)					
<3	191(47.9)	150(55.6)	41(31.8)	136(48.6)	55(46.2)
≥3	208(52.1)	120(44.4)	88(68.2)	144(51.4)	64(53.8)
Differentiation Grad					
Well	25(6.3)	23(8.5)	2(1.6)	20(7.1)	5(4.2)
Moderately	186(46.6)	136(50.4)	50(38.8)	129(46.1)	57(47.9)
Poorly	188(47.1)	111(41.1)	77(59.7)	131(46.8)	57(47.9)
LVSI					
No	345(86.5)	246(91.1)	99(76.7)	242(86.4)	103(86.6)
Yes	54(13.5)	24(8.9)	30(23.3)	38(13.6)	16(13.4)
Perineuronal invasion					
No	350(87.7)	245(90.7)	105(81.4)	248(88.6)	102(85.7)
Yes	49(12.3)	25(9.3)	24(18.6)	32(11.4)	17(14.3)
LNR(%)					
<12	338(84.7)	245(90.7)	93(72.1)	241(86.1)	97(81.5)
≥12	61(15.3)	25(9.3)	36(27.9)	39(13.9)	22(18.5)
Surgery approach					
Sweet	30(7.5)	20(7.4)	10(7.8)	19(6.8)	19(16.0)
Ivor Lewis	302(75.7)	202(74.8)	100(77.5)	215(76.8)	215(180.7)
McKeown	67(16.8)	48(17.8)	19(14.7)	46(16.4)	46(38.7)
pT stage					
1	138(34.6)	125(46.3)	13(10.1)	99(35.4)	39(32.8)
2	79(19.8)	57(21.1)	22(17.1)	55(19.6)	24(20.2)
3	182(45.6)	88(32.6)	94(72.9)	126(45.0)	56(47.1)
pN stage					
0	234(58.6)	205(75.9)	29(22.5)	166(59.3)	68(57.1)
1	135(33.8)	55(20.4)	80(62.0)	94(33.6)	41(34.5)
2	30(7.5)	10(3.7)	20(15.5)	20(7.1)	10(8.4)
pTNM stage					
I	116(29.1)	116(43.0)	0(0)	84(30.0)	32(26.9)
II	141(35.3)	99(36.7)	42(32.6)	98(35.0)	43(36.1)
III	142(35.6)	55(20.4)	87(67.4)	98(35.0)	44(37.0)

### Independent prognostic factors for OS of patients

**Fig. 2:**
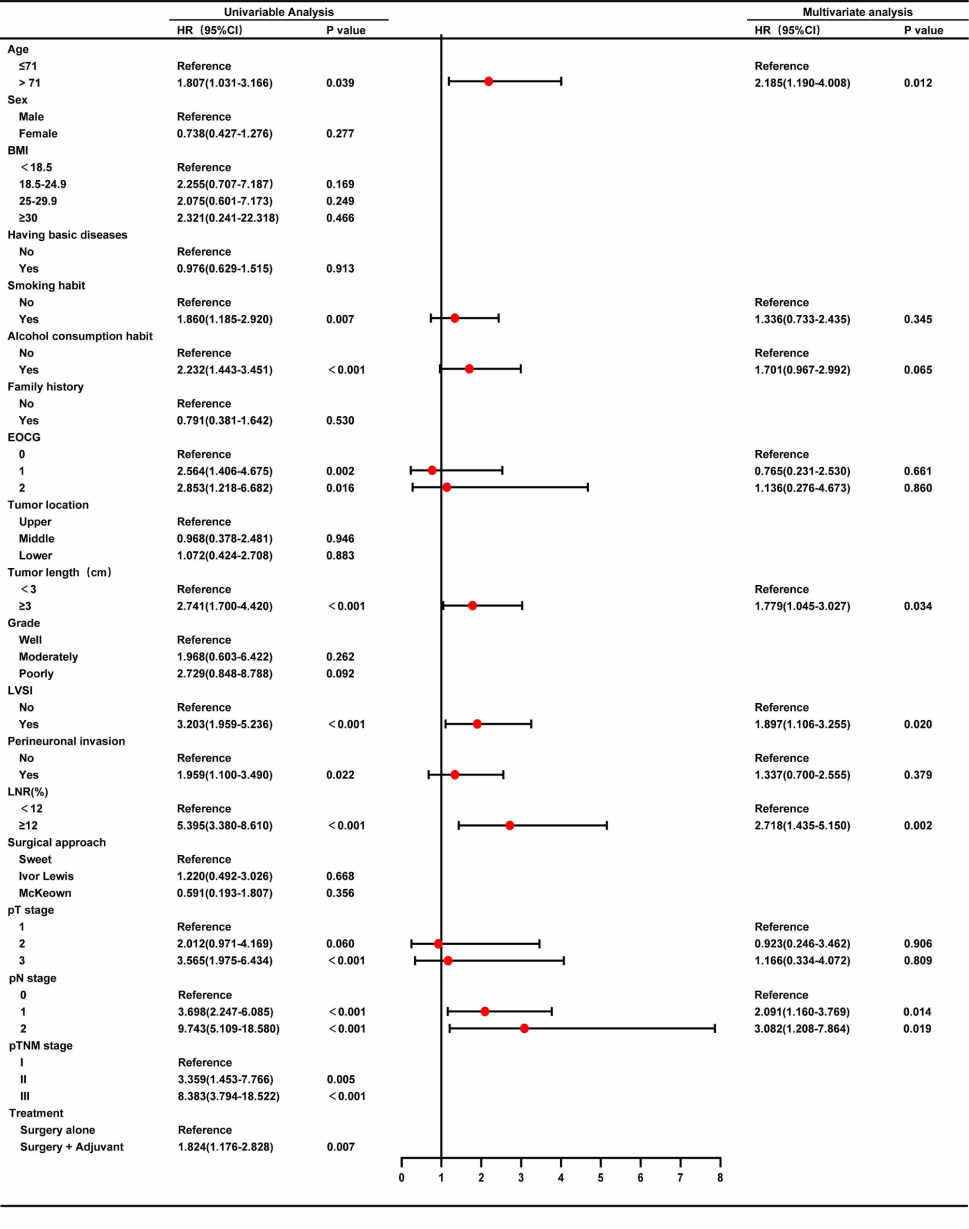
Forest plot of the univariate and multivariate analysis by Cox proportional hazards model in the training cohort

Univariate Cox analysis was performed on the training cohort and identified 10 significant prognostic factors for OS: age, smoking habit, alcohol consumption habit, ECOG PS score, tumor length, LVSI, perineuronal invasion, LNR, pathological T stage, pathological N stage. These aforementioned factors were further analyzed in a multivariate Cox regression analysis, which revealed that age (*p* = 0.012, HR = 2.185), tumor length (*p* = 0.034, HR = 1.779), LVSI (*p* = 0.020, HR = 1.897), LNR (*p* = 0.002, HR = 2.718), and pathological N stage (N1: *p* = 0.014, HR = 2.091; N2: *p* = 0.019, HR = 3.082) were independent prognostic factors for OS (Fig. 2).

### Prognostic nomogram construction and external validation

**Fig. 3:**
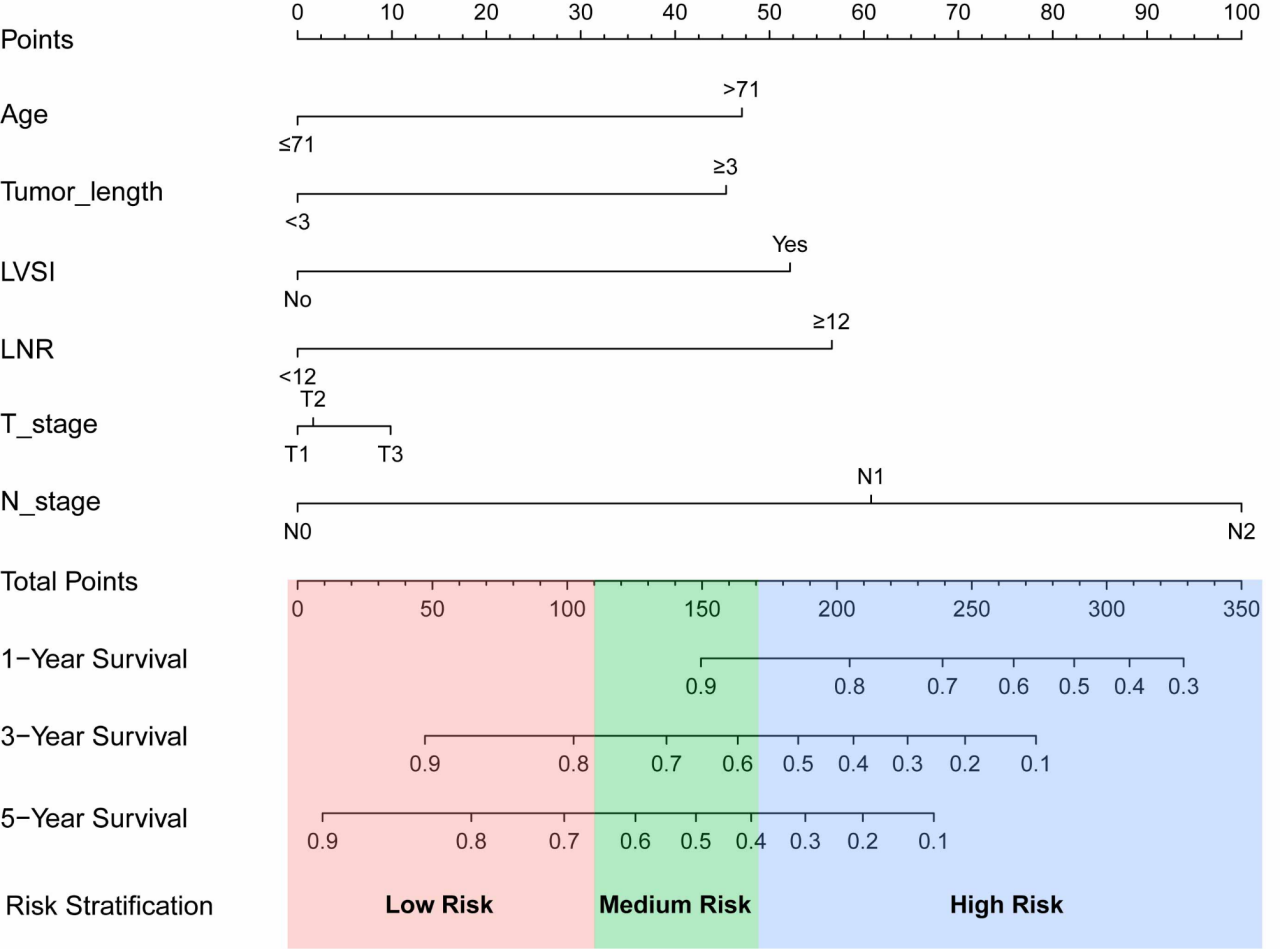
Prognostic nomogram for ESCC patients who receive surgery

Based on the discovery of five autonomous prognostic factors through univariate and multivariate Cox analyses, a nomogram was constructed for prognosticating the outcome of ESCC patients following radical surgery. Considering that the pathological T stage holds significant recognition as an autonomous prognostic factor for esophageal squamous cell carcinoma (ESCC) and serves as a guiding principle in its clinical management, it was duly incorporated into the nomogram model. As demonstrated in Fig. 3, the pathological N stage exhibited the most pronounced impact on survival outcomes, followed by lymph node ratio (LNR), LVSI, age, tumor length, and pathological T stage. Each patient’s clinical and pathological characteristics were assigned corresponding scores based on the nomogram’s first row (Points), which were summed to calculate a total score. Patients’ 1-year, 3-year, and 5-year survival rates were estimated on the survival scale by drawing a vertical line on the basis of the Total Points.

**Fig. 4:**
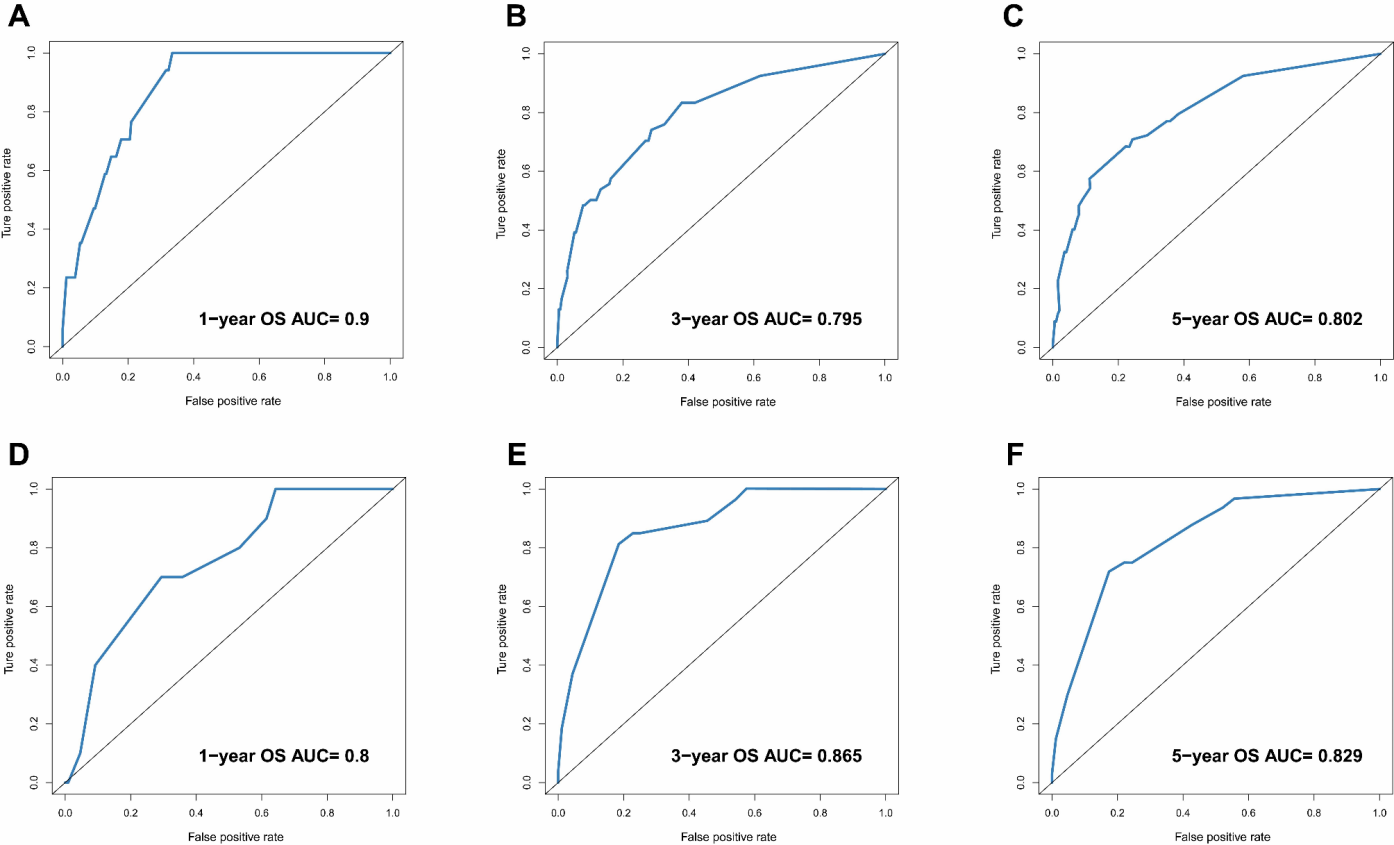
ROC curves to predict patient survival. (**A**) at 1-year OS in the training cohort; (**B**) at 3-year OS in the training cohort; (**C**) at 5-year OS in the training cohort; (**D**) at 1-year OS in the external validation cohort; (**E**) at 3-year OS in the validation cohort; (**F**) at 5-year OS in the validation cohort

As the time-dependent ROC curves analysis illustrated in Fig. 4, the area under the ROC curve (AUC) values for the 1-, 3-, and 5-year OS rates of the training cohort were 0.900, 0.795, and 0.802, respectively, while the AUC values for the 1-, 3-, and 5-year OS rates of the external validation cohort were 0.800, 0.865, 0.829, respectively. In the case of AUC ≥ 0.5, the closer the AUC value is to 1, the better the discrimination ability. Therefore, the result above demonstrated a good survival prediction performance.

**Fig. 5:**
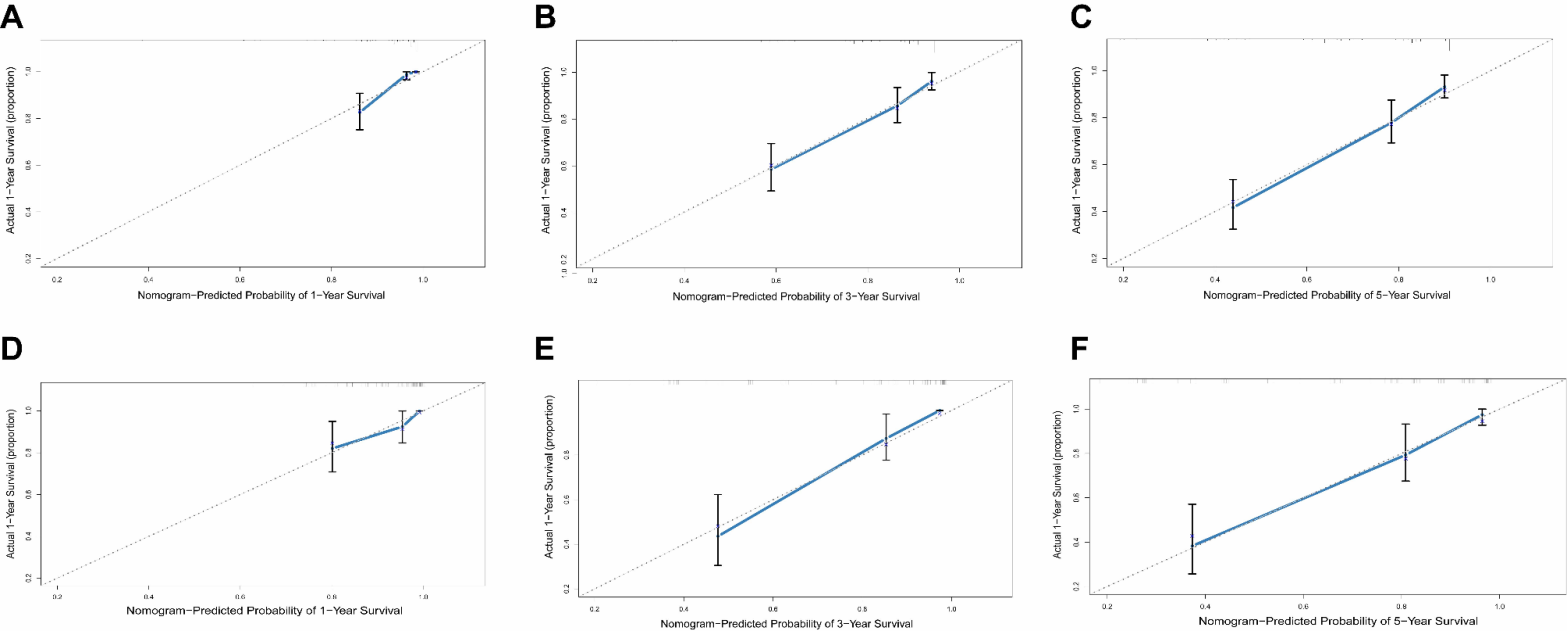
Calibration curves to predict patient survival. (**A**) at 1-year in the training cohort; (**B**) at 3-year in the training cohort; (**C**) at 5-year in the training cohort; (**D**) at 1-year OS in the external validation cohort; (**E**) at 3-year in the validation cohort; (**F**) at 5-year in the validation cohort. Nomogram-predicted probability of overall survival is displayed on the x-axis; the y-axis refers to the actual overall survival; the gray dotted line corresponds to a 45-degree reference line

Furthermore, the calibration curves demonstrated remarkable consistency between the projected and observed 1-year, 3-year, and 5-year OS rates both in the training cohort and external validation cohort (Fig. 5**)**. These findings suggest that the nomogram model has commendable precision and dependability for predicting postoperative prognosis in ESCC patients.

### Comparison between the prognostic nomogram with the AJCC 8th TNM staging system

**Fig. 6:**
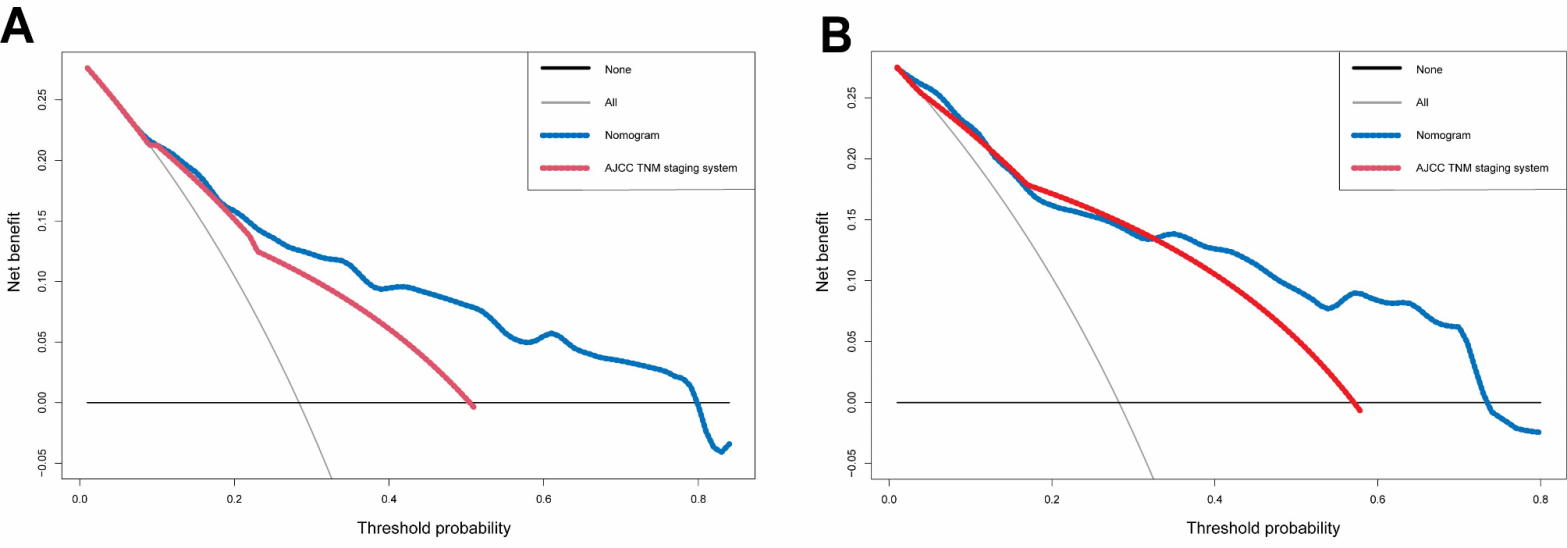
Decision curve analysis of the nomogram (blue line) and the AJCC 8th TNM staging system (red line) for 5-year OS prediction. (**A**) in the training cohort; (**B**) in the validation cohort

The nomogram model demonstrated significantly improved accuracy over the AJCC 8th TNM staging system, with a higher C-index value of 0.769(95% CI: 0.744–0.794) compared to 0.708 (95% CI: 0.684–0.732), indicating a significant increase of 0.061 (*p* < 0.001) in the C-index value. Furthermore, the DCA demonstrated that the nomogram exhibited enhanced clinical utility. Compared to the AJCC 8th TNM staging system, the nomogram manifesting a superior net benefit throughout nearly the entire range of threshold probabilities, as delineated in Fig. 6.

### Risk stratification and subgroup analysis based on the Nomogram

**Fig. 7:**
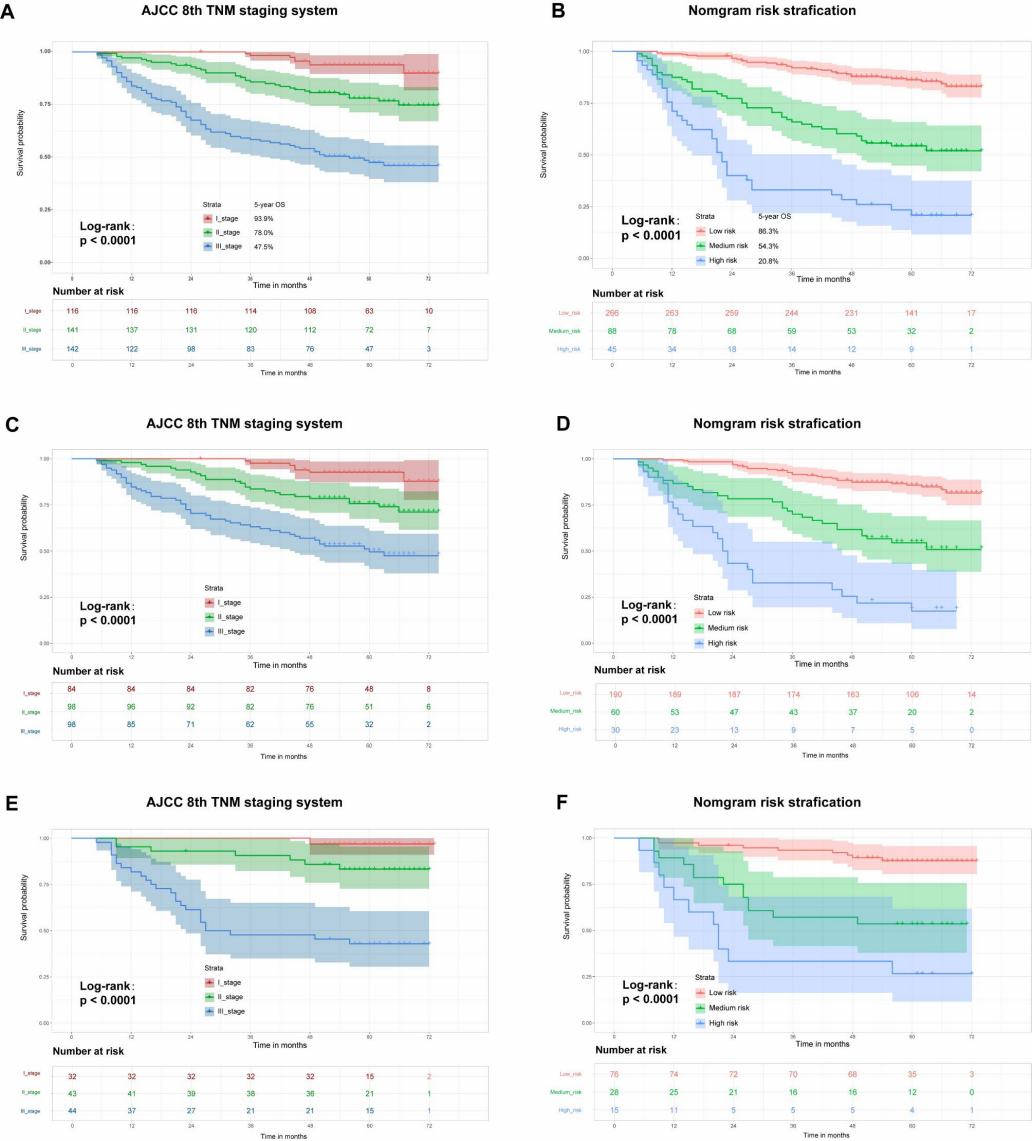
Kaplan-Meier curve based on AJCC 8th TNM staging and nomogram risk stratification. (**A, B**) in the entire cohort; (**C, D**) in the training cohort; (**E, F**) in the validation cohort

Risk stratification was conducted for a total of 399 patients diagnosed with ESCC based on the computed overall score obtained from the nomogram. As indicated in the nomogram (Fig. 3), these patients were categorized into three groups: low-risk (0-110.9 points), intermediate-risk (111-171.9 points), and high-risk (172-311.1 points). Kaplan-Meier analysis showed statistically significant differences in OS among the three risk groups (*p* < 0.0001) (Fig. 7B). In the overall population, the 1-year OS rates for the low-, intermediate-, and high-risk groups were 98.9%, 92.8%, and 86.3%, respectively; the 3-year OS rates were 87.5%, 65.9%, and 54.3%, respectively; and the 5-year OS rates were75.6%, 33.1%, and 20.8%, respectively. In comparison, survival analysis according to the AJCC 8th TNM staging system showed that the 1-year OS rates for patients with ESCC in stages I, II, and III were 100%, 97.2%, and 83.8%, respectively; the 3-year OS rates were 98.3%, 85.7%, and 58.4%, respectively; and the 5-year OS rates were 93.9%, 78.0%, and 47.5%, respectively. (Fig. 7A). These findings suggest that risk stratification based on the nomogram may identify low, intermediate, and high-risk patients, providing additional information beyond that offered by the AJCC 8th TNM staging system. The similar results were also confirmed in the training cohort and the validation cohort (Fig. 7C, D, E and F).

### The impact of adjuvant therapy on OS of postoperative patients with ESCC

**Fig. 8:**
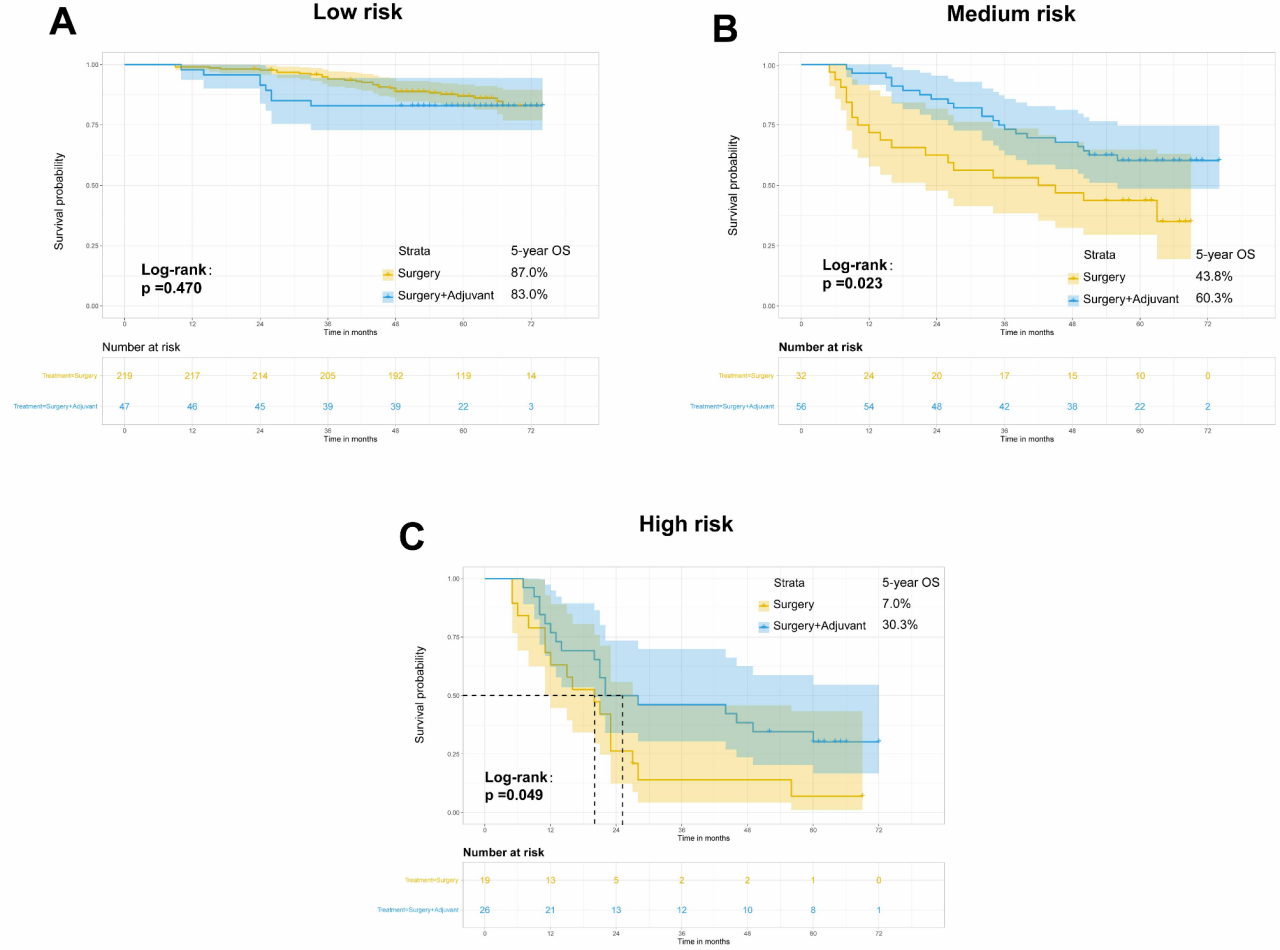
Subgroup survival analysis of patients with ESCC after radical surgery

The aim of this study was to examine the effect of postoperative adjuvant therapy on OS among different risk groups of ESCC patients stratified by the nomogram. In the low-risk group, no statistically significant disparity was observed in OS between the pure surgery group and the postoperative adjuvant therapy group (*p* = 0.470) (Fig. 8A). In the medium-risk group, the adjuvant therapy cohort exhibited an approximately 16.5% increase in 5-year OS rates in comparison with the surgery-alone group (*p* = 0.023) (Fig. 8B). Among the individuals categorized as high-risk, the median OS of patients receiving surgery alone was 20 months, while that for patients receiving postoperative adjuvant therapy was 25 months. The group that underwent postoperative adjuvant therapy exhibited notably elevated OS rates in comparison to the group that solely underwent surgery (*p* = 0.049). (Fig. 8C). These results suggested that postoperative adjuvant therapy has the potential to significantly extend the lifetime of ESCC patients who are classified as high-risk, with a median survival extension of 5 months.

## Discussion

Esophageal cancer is characterized by high incidence, poor prognosis, and low treatment efficacy. Currently, surgical resection remains the mainstay treatment for operable esophageal cancer, but it is important to note that patients who undergo radical surgery alone face a significant risk of recurrence and metastasis [29]. Therefore, exploring comprehensive treatment strategies for operable esophageal cancer has become a research hotspot. Based on the CROSS study, neoadjuvant chemoradiotherapy combined with surgery is recommended as the standard treatment for EAC in Western countries [30]. However, in China, where ESCC is the predominant subtype, a considerable number of patients undergo radical surgery without prior administration of neoadjuvant therapy. At present, the global medical community is still grappling with the question of whether postoperative adjuvant therapy can bring advantages to patients who have undergone radical surgery for ESCC. Consequently, the identification of potential recipients has emerged as a crucial matter that demands immediate attention. In this study, a nomogram was developed to predict the 1-, 3-, and 5-year OS for stage I-III ESCC patients who underwent R0 surgery. The nomogram demonstrated superior dependability, precision, and practicality in comparison to the AJCC 8th TNM staging system. By utilizing the nomogram, patients were categorized into three risk groups with corresponding prognostic scores. Our findings suggested that postoperative adjuvant therapy can significantly improve OS for medium-risk and high-risk ESCC patients.

The findings of this study, which involved 399 patients with stage I-III ESCC who underwent complete resection with or without postoperative adjuvant radiotherapy/chemoradiotherapy, revealed that age, tumor length, LVSI, LNR, and pathological N stage were identified as independent prognostic factors through Cox regression analysis. These results align with previous research conducted on the subject. Firstly, age has been found to have a notable correlation with the prognosis of ESCC [31]. This relationship may be attributed to metabolic disorders and ailments that arise as a consequence of the aging process [32]. Some studies have suggested that postoperative adjuvant radiotherapy may confer survival benefits for elderly patients with thoracic ESCC [33]. In addition, the length of the tumor is a crucial determinant in the prognosis of individuals diagnosed with esophageal cancer. By employing X-tile software, this investigation determined the cutoff point for tumor length to be 3 cm. It was discovered that patients with tumors exceeding this threshold exhibited a significantly inferior prognosis, aligning with prior research findings [34, 35]. Due to the tendency of ESCC cells to grow longitudinally on mucosa rich in lymphatic vessels, tumor length can indicate possible peritumoral micrometastasis or even lymph node metastasis when it exceeds a certain length [36]. LVSI has demonstrated unfavorable prognostic implications in diverse cancers such as lung cancer, bladder cancer, and endometrial cancer [37–39]. Similar findings have been found in ESCC, where LVSI is associated with high invasiveness and an increased risk of tumor metastasis, particularly in lymph node-negative patients after esophageal cancer surgery [40–42]. Furthermore, LNR is also an important prognostic factor for postoperative ESCC, with patients having an LNR greater than 12% having a poorer prognosis, which is consistent with similar reports in multiple studies [43–47]. Ensuring adequate clearance of lymph nodes during radical surgery for ESCC is challenging due to various factors, including the patient’s physical condition, pathological diagnosis, and surgical conditions. Therefore, LNR is considered a supplement to the lymph node classification in the AJCC TNM staging system [48]. Nevertheless, several studies have indicated that LNR cannot serve as a full substitute for the count of metastatic lymph nodes when determining the pN staging in ESCC [49]. Therefore, this study included both LNR and pN staging in the line graph prediction model, which is similar to other studies that also included these two variables representing lymph node status in the model [46]. In addition, multiple studies have demonstrated that postoperative adjuvant radiotherapy/chemoradiotherapy can effectively extend the lifespan of patients with pN+ [22]. Similar to pathological N staging, pathological T staging is also a critical element impacting the survival of postoperative esophageal cancer patients. Although the results of the multivariate analysis were not ideal, the univariate analysis demonstrated a significant association between pathological T staging and OS in ESCC patients after surgery. The later the T staging, the worse the prognosis, which has also been observed in previous studies [10, 50]. Previous studies have also shown that patients with T3-4 ESCC after radical surgery have a poor prognosis [51, 52], and that patients with T3 ESCC may benefit from postoperative adjuvant therapy in terms of survival [16, 18].

This study developed a model for postoperative ESCC based on Cox regression analysis, which performed well in external validation. To our knowledge, there are currently no studies reporting a prediction model with a C-index value exceeding 0.750 for postoperative OS in esophageal cancer [42, 46, 47, 50–57]. However, the model formulated in this research exhibited a commendable C-index value of 0.769 and an AUC value almost surpassing 0.80, indicating its exceptional precision. The calibration curves for the 1-year, 3-year, and 5-year OS-related prediction models also exhibited commendable consistency. Additionally, the DCA curve indicated that the prediction model developed in this study had superior clinical predictive value in comparison to the AJCC 8th TNM staging system.

Although postoperative adjuvant therapy for ESCC remains controversial, previous research has shown that postoperative adjuvant radiotherapy/chemoradiotherapy can enhance survival rates and reduce recurrence in patients with stage IIB-III ESCC after surgery [11–15]. To further select the population who benefit from postoperative adjuvant therapy, risk stratification was performed on the overall population who received or did not receive postoperative adjuvant radiotherapy/chemoradiotherapy according to the ESCC postoperative nomogram model, and survival analysis was conducted. It was found that the 5-year survival rates of the intermediate- and high-risk groups who received postoperative adjuvant therapy increased by 16.5% and 23.3%, respectively. In other words, ESCC patients with a total score exceeding 111 in the line graph model may benefit from postoperative adjuvant therapy. This research applied the established model to identify potential beneficiaries of postoperative adjuvant therapy, providing a valuable reference for developing personalized treatment strategies for postoperative ESCC patients in clinical practice.

In the course of conducting this research, certain restrictions were encountered that should be acknowledged. Firstly, it is a retrospective analysis featuring a limited sample size, thus being inevitably affected by potential confounding factors and selection bias. Secondly, this nomogram model was constructed using clinical data solely from a single center without external validation, thus its performance needs to be tested and generalized in larger patient cohorts from diverse geographical regions. Thirdly, the variables included in this study were confined to clinical-pathological features. Subsequent investigations will explore the impact of innovative features such as hematological, radiomic, and genomic traits on the survival of postoperative ESCC patients [58–60]. More extensive and precise predictive models will be built to provide more dependable guidance for individualized diagnosis and treatment of postoperative ESCC patients. Ultimately, data correlated with survival outcomes, such as postoperative complications, chemotherapy regimens, adverse reactions, patient compliance, and other pertinent factors, were not accessible. Consequently, we assert that further prospective studies and well-designed clinical trials are needed to identify the treatment options with the greatest benefits.

To summarize and conclude, this research identified age, tumor length, LVSI, LNR, and pathological N stage as independent prognostic factors for patients undergoing radical surgery for ESCC. A nomogram model was developed for individualized decision-making on postoperative adjuvant therapy in I-III stage ESCC patients who have received R0 resection, with clinical utility superior to the AJCC 8th TNM staging system. Based on our findings, we recommend the administration of postoperative adjuvant therapy for patients at high-risk and medium-risk.

## Electronic supplementary material

Below is the link to the electronic supplementary material.

Supplementary Material 1

Supplementary Material 2

## Data Availability

The datasets used and/or analysed during the current study available from the corresponding author on reasonable request.

## References

[1] Sung H, Ferlay J, Siegel RL, Laversanne M, Soerjomataram I, Jemal A, Bray F. Global Cancer statistics 2020: GLOBOCAN estimates of incidence and Mortality Worldwide for 36 cancers in 185 countries. CA Cancer J Clin. 2021;71(3):209–49. 33538338 10.3322/caac.21660

[2] Feng RM, Zong YN, Cao SM, Xu RH. Current cancer situation in China: good or bad news from the 2018 Global Cancer statistics? Cancer Commun (Lond), 2019;39(1):22. 10.1186/s40880-019-0368-6 PMC6487510 31030667

[3] Abnet CC, Arnold M, Wei WQ. Epidemiology of esophageal squamous cell carcinoma. Gastroenterology. 2018;154(2):360–73. 28823862 10.1053/j.gastro.2017.08.023 PMC5836473

[4] Reichenbach ZW, Murray MG, Saxena R, Farkas D, Karassik EG, Klochkova A, Patel K, Tice C, Hall TM, Gang J, Parkman HP, Ward SJ, Tetreault MP, Whelan KA. Clinical and translational advances in esophageal squamous cell carcinoma. Adv Cancer Res. 2019;144:95–135. 31349905 10.1016/bs.acr.2019.05.004

[5] Cui Y, Chen H, Xi R, Cui H, Zhao Y, Xu E, Yan T, Lu X, Huang F, Kong P, Li Y, Zhu X, Wang J, Zhu W, Wang J, Ma Y, Zhou Y, Guo S, Zhang L, Liu Y, Wang B, Xi Y, Sun R, Yu X, Zhai Y, Wang F, Yang J, Yang B, Cheng C, Liu J, Song B, Li H, Wang Y, Zhang Y, Cheng X, Zhan Q, Li Y, Liu Z. Whole-genome sequencing of 508 patients identifies key molecular features associated with poor prognosis in esophageal squamous cell carcinoma. Cell Res. 2020;30(10):902–13. 32398863 10.1038/s41422-020-0333-6 PMC7608103

[6] Gronnier C, Collet D. New Trends in Esophageal Cancer Management. Cancers (Basel), 2021,13(12). 10.3390/cancers13123030 PMC8235022 34204314

[7] Altorki N, Skinner D. Should en bloc esophagectomy be the standard of care for esophageal carcinoma? Ann Surg. 2001;234(5):581–7. 11685019 10.1097/00000658-200111000-00001 PMC1422081

[8] Oshikiri T, Numasaki H, Oguma J, Toh Y, Watanabe M, Muto M, Kakeji Y, Doki Y. Prognosis of patients with Esophageal Carcinoma following routine thoracic Duct Resection: a propensity-matched analysis of 12,237 patients based on the Comprehensive Registry of Esophageal Cancer in Japan. Ann Surg, 2021. 10.1097/SLA.0000000000005340 34913902

[9] Kanda M, Koike M, Shimizu D, Tanaka C, Hattori N, Hayashi M, Yamada S, Omae K, Kodera Y. Characteristics Associated with nodal and distant recurrence after radical esophagectomy for squamous cell carcinoma of the thoracic esophagus. Ann Surg Oncol. 2020;27(9):3195–205. 32246314 10.1245/s10434-020-08433-6

[10] Chang X, Chen J, Zhang W, Yang J, Yu S, Deng W, Ni W, Zhou Z, Chen D, Feng Q, Lv J, Liang J, Hui Z, Wang L, Lin Y, Chen X, Xue Q, Mao Y, Gao Y, Wang D, Feng F, Gao S, He J, Xiao Z. Recurrence risk stratification based on a competing-risks nomogram to identify patients with esophageal cancer who may benefit from postoperative radiotherapy. Ther Adv Med Oncol. 2021;13:17588359211061948. 34987617 10.1177/17588359211061948 PMC8721393

[11] Yu S, Zhang W, Ni W, Xiao Z, Wang Q, Zhou Z, Feng Q, Zhang H, Chen D, Liang J, Lv J, Hui Z, He J, Gao S, Sun K, Fang D, Liu X, Li Y. A propensity-score matching analysis comparing long-term survival of surgery alone and postoperative treatment for patients in node positive or stage III esophageal squamous cell carcinoma after R0 esophagectomy. Radiother Oncol. 2019;140:159–66. 31302346 10.1016/j.radonc.2019.06.020

[12] Zou B, Pang J, Liu Y, Xu Y, Li L, Zhou L, Zhu J, Huang M, Wang J, Ren L, Gong Y, Lu Y, Chen L, Peng F. Postoperative chemoradiotherapy improves survival in patients with stage II-III esophageal squamous cell carcinoma: an analysis of clinical outcomes. Thorac Cancer. 2016;7(5):515–21. 27766781 10.1111/1759-7714.12355 PMC5129165

[13] Zou B, Tu Y, Liao D, Xu Y, Wang J, Huang M, Ren L, Zhu J, Gong Y, Liu Y, Zhou L, Zhou X, Peng F, Lu Y. Radical esophagectomy for stage II and III thoracic esophageal squamous cell carcinoma followed by adjuvant radiotherapy with or without chemotherapy: which is more beneficial? Thorac Cancer. 2020;11(3):631–9. 31943824 10.1111/1759-7714.13307 PMC7049519

[14] Ni W, Yu S, Zhang W, Xiao Z, Zhou Z, Chen D, Feng Q, Liang J, Lv J, Gao S, Mao Y, Xue Q, Sun K, Liu X, Fang D, Li J, Wang D. A phase-II/III randomized controlled trial of adjuvant radiotherapy or concurrent chemoradiotherapy after surgery versus surgery alone in patients with stage-IIB/III esophageal squamous cell carcinoma. BMC Cancer. 2020;20(1):130. 32070309 10.1186/s12885-020-6592-2 PMC7027054

[15] Ni W, Yu S, Xiao Z, Zhou Z, Chen D, Feng Q, Liang J, Lv J, Gao S, Mao Y, Xue Q, Sun K, Liu X, Fang D, Li J, Wang D, Zhao J, Gao Y. Postoperative adjuvant therapy versus surgery alone for stage IIB-III esophageal squamous cell carcinoma: a phase III Randomized Controlled Trial. Oncologist. 2021;26(12):e2151–60. 34309117 10.1002/onco.13914 PMC8649038

[16] Shen WB, Gao HM, Zhu SC, Li YM, Li SG, Xu JR. Analysis of the causes of failure after radical surgery in patients with PT3N0M0 thoracic esophageal squamous cell carcinoma and consideration of postoperative radiotherapy. World J Surg Oncol. 2017;15(1):192. 29070049 10.1186/s12957-017-1259-4 PMC5657067

[17] Yang J, Zhang W, Xiao Z, Wang Q, Zhou Z, Zhang H, Chen D, Feng Q, He J, Gao S, Sun K, Liu X, Fang D, Mu J, Wang D, Li Y. The Impact of Postoperative Conformal Radiotherapy after radical surgery on survival and recurrence in pathologic T3N0M0 esophageal carcinoma: a propensity score-matched analysis. J Thorac Oncol. 2017;12(7):1143–51. 28411098 10.1016/j.jtho.2017.03.024

[18] Chen SB, Weng HR, Wang G, Liu DT, Li H, Zhang H, Chen YP. The impact of adjuvant radiotherapy on radically resected T3 esophageal squamous cell carcinoma. J Cancer Res Clin Oncol. 2016;142(1):277–86. 26328915 10.1007/s00432-015-2041-z PMC11819054

[19] Deng W, Yang J, Ni W, Li C, Chang X, Han W, Zhou Z, Chen D, Feng Q, Liang J, Lv J, Wang X, Wang X, Deng L, Wang W, Bi N, Zhang T, Li Y, Gao S, Xue Q, Mao Y, Sun K, Liu X, Fang D, Wang D, Li J, Zhao J, Shao K, Li Z, Chen X, Han L, Wang L, He J, Xiao Z. Postoperative Radiotherapy in pathological T2-3N0M0 thoracic esophageal squamous cell carcinoma: Interim Report of a prospective, phase III, Randomized Controlled Study. Oncologist. 2020;25(4):e701–8. 32083766 10.1634/theoncologist.2019-0276 PMC7160414

[20] Kam AE, Pappas SG, Masood A. Postoperative chemotherapy for thoracic pathological T3N0M0 esophageal squamous cell carcinoma. Ann Surg Oncol. 2020;27(5):1314–5. 31848823 10.1245/s10434-019-08140-x

[21] He M, Qi Z, Qiu R, Hu Y, Li J, Li Y, Wang Y. Correlates of long-term survival of patients with pN + esophageal squamous cell carcinoma after Esophagectomy. J Oncol. 2021;2021:6675691. 33679976 10.1155/2021/6675691 PMC7906819

[22] Li J, Qiu R, Hu Y, Wang Y, Qi Z, He M, Li Y. Postoperative adjuvant therapy for patients with pN + esophageal squamous cell carcinoma. Biomed Res Int. 2021;2021:8571438. 33553432 10.1155/2021/8571438 PMC7847342

[23] Hwang JY, Chen HS, Hsu PK, Chao YK, Wang BY, Huang CS, Liu CC, Wu SC. A propensity-matched analysis comparing Survival after Esophagectomy followed by adjuvant chemoradiation to surgery alone for esophageal squamous cell carcinoma. Ann Surg. 2016;264(1):100–6. 26649580 10.1097/SLA.0000000000001410

[24] Amin MB, Greene FL, Edge SB, Compton CC, Gershenwald JE, Brookland RK, Meyer L, Gress DM, Byrd DR, Winchester DP. The Eighth Edition AJCC Cancer staging Manual: continuing to build a bridge from a population-based to a more personalized approach to cancer staging. CA Cancer J Clin. 2017;67(2):93–9. 28094848 10.3322/caac.21388

[25] Iasonos A, Schrag D, Raj GV, Panageas KS. How to build and interpret a nomogram for cancer prognosis. J Clin Oncol. 2008;26(8):1364–70. 18323559 10.1200/JCO.2007.12.9791

[26] Balachandran VP, Gonen M, Smith JJ, DeMatteo RP. Nomograms in oncology: more than meets the eye. Lancet Oncol. 2015;16(4):e173–80. 25846097 10.1016/S1470-2045(14)71116-7 PMC4465353

[27] Camp RL, Dolled-Filhart M, Rimm DL. X-tile: a new bio-informatics tool for biomarker assessment and outcome-based cut-point optimization. Clin Cancer Res. 2004;10(21):7252–9. 15534099 10.1158/1078-0432.CCR-04-0713

[28] Fitzgerald M, Saville BR, Lewis RJ. Decision curve analysis. JAMA. 2015;313(4):409–10. 25626037 10.1001/jama.2015.37

[29] Chen G, Wang Z, Liu XY, Liu FY. Recurrence pattern of squamous cell carcinoma in the middle thoracic esophagus after modified Ivor-Lewis esophagectomy. World J Surg. 2007;31(5):1107–14. 17426905 10.1007/s00268-006-0551-1

[30] Shapiro J, van Lanschot JJB, Hulshof M, van Hagen P, van Berge Henegouwen MI, Wijnhoven BPL, van Laarhoven HWM, Nieuwenhuijzen GAP, Hospers GAP, Bonenkamp JJ, Cuesta MA, Blaisse RJB, Busch ORC, Ten Kate FJW, Creemers GM, Punt CJA, Plukker JTM, Verheul HMW, Bilgen EJS, van Dekken H, van der Sangen MJC, Rozema T, Biermann K, Beukema JC, Piet AHM, van Rij CM, Reinders JG, Tilanus HW, Steyerberg EW, van der Gaast A, group Cs. Neoadjuvant chemoradiotherapy plus surgery versus surgery alone for oesophageal or junctional cancer (CROSS): long-term results of a randomised controlled trial. Lancet Oncol, 2015;16(9):1090–8. 10.1016/S1470-2045(15)00040-6 26254683

[31] Baranov NS, Slootmans C, van Workum F, Klarenbeek BR, Schoon Y, Rosman C. Outcomes of curative esophageal cancer surgery in elderly: a meta-analysis. World J Gastrointest Oncol. 2021;13(2):131–46. 33643529 10.4251/wjgo.v13.i2.131 PMC7896422

[32] Mantziari S, Teixeira Farinha H, Bouygues V, Vignal JC, Deswysen Y, Demartines N, Schafer M, Piessen G. Esophageal Cancer in Elderly Patients, Current Treatment Options and Outcomes; A Systematic Review and Pooled Analysis. Cancers (Basel), 2021;13(9). 10.3390/cancers13092104 PMC8123886 33925512

[33] Jiang W, Sun X, Zhou B, Han C, Liu F, Zheng J, Sun X. Evaluation of surgery plus postoperative radiotherapy or definitive radiotherapy in older patients with thoracic esophageal squamous cell cancer. J Cancer Res Ther. 2019;15(4):849–56. 31436242 10.4103/jcrt.JCRT_789_18

[34] Wang BY, Goan YG, Hsu PK, Hsu WH, Wu YC. Tumor length as a prognostic factor in esophageal squamous cell carcinoma. Ann Thorac Surg. 2011;91(3):887–93. 21353021 10.1016/j.athoracsur.2010.11.011

[35] Hollis AC, Quinn LM, Hodson J, Evans E, Plowright J, Begum R, Mitchell H, Hallissey MT, Whiting JL, Griffiths EA. Prognostic significance of tumor length in patients receiving esophagectomy for esophageal cancer. J Surg Oncol. 2017;116(8):1114–22. 28767142 10.1002/jso.24789

[36] Yendamuri S, Swisher SG, Correa AM, Hofstetter W, Ajani JA, Francis A, Maru D, Mehran RJ, Rice DC, Roth JA, Walsh GL, Vaporciyan AA. Esophageal tumor length is independently associated with long-term survival. Cancer. 2009;115(3):508–16. 19117343 10.1002/cncr.24062

[37] Raffone A, Travaglino A, Raimondo D, Neola D, Maletta M, Santoro A, Insabato L, Casadio P, Fanfani F, Zannoni GF, Zullo F, Seracchioli R, Mollo A. Lymphovascular space invasion in endometrial carcinoma: a prognostic factor independent from molecular signature. Gynecol Oncol, 2022. 10.1016/j.ygyno.2022.01.013 35078650

[38] Mathieu R, Lucca I, Roupret M, Briganti A, Shariat SF. The prognostic role of lymphovascular invasion in urothelial carcinoma of the bladder. Nat Rev Urol. 2016;13(8):471–9. 27431340 10.1038/nrurol.2016.126

[39] Mollberg NM, Bennette C, Howell E, Backhus L, Devine B, Ferguson MK. Lymphovascular invasion as a prognostic indicator in stage I non-small cell lung cancer: a systematic review and meta-analysis. Ann Thorac Surg. 2014;97(3):965–71. 24424014 10.1016/j.athoracsur.2013.11.002

[40] Hsu CP, Chuang CY, Hsu PK, Chien LI, Lin CH, Yeh YC, Hsu HS, Wu YC. Lymphovascular Invasion as the major prognostic factor in node-negative esophageal Cancer after primary esophagectomy. J Gastrointest Surg. 2020;24(7):1459–68. 31273552 10.1007/s11605-019-04310-0

[41] Chen WH, Huang YL, Chao YK, Yeh CJ, Chang HK, Tseng CK, Liu YH. Prognostic significance of lymphovascular invasion in patients with esophageal squamous cell carcinoma treated with neoadjuvant chemoradiotherapy. Ann Surg Oncol. 2015;22(1):338–43. 25023545 10.1245/s10434-014-3881-5

[42] Deng W, Zhang W, Yang J, Ni W, Yu S, Li C, Chang X, Zhou Z, Chen D, Feng Q, Chen X, Lin Y, Zhu K, Zheng X, He J, Gao S, Xue Q, Mao Y, Cheng G, Sun K, Liu X, Fang D, Chen J, Xiao Z. Nomogram to predict overall survival for thoracic esophageal squamous cell Carcinoma patients after Radical Esophagectomy. Ann Surg Oncol. 2019;26(9):2890–8. 31183641 10.1245/s10434-019-07393-w

[43] Hou X, Wei JC, Xu Y, Luo RZ, Fu JH, Zhang LJ, Lin P, Yang HX. The positive lymph node ratio predicts long-term survival in patients with operable thoracic esophageal squamous cell carcinoma in China. Ann Surg Oncol. 2013;20(5):1653–9. 23247981 10.1245/s10434-012-2794-4

[44] Yukawa N, Aoyama T, Tamagawa H, Tamagawa A, Atsumi Y, Kawahara S, Maezawa Y, Kano K, Murakawa M, Kazama K, Numata M, Oshima T, Masuda M, Rino Y. The lymph node ratio is an independent prognostic factor in Esophageal Cancer patients who receive curative surgery. Vivo. 2020;34(4):2087–93. 10.21873/invivo.12012 PMC7439888 32606187

[45] Liu YP, Ma L, Wang SJ, Chen YN, Wu GX, Han M, Wang XL. Prognostic value of lymph node metastases and lymph node ratio in esophageal squamous cell carcinoma. Eur J Surg Oncol. 2010;36(2):155–9. 19854606 10.1016/j.ejso.2009.09.005

[46] Zhao Z, Huang X, Gu T, Chen Z, Gan L, Zhu B, Wu N. Predicting Individual Survival after Curative Esophagectomy for Squamous Cell Carcinoma of Esophageal. Gastroenterol Res Pract. 2021;2021:5595718. 33883995 10.1155/2021/5595718 PMC8041542

[47] Lin MQ, Li JL, Zhang ZK, Chen XH, Ma JY, Dai YQ, Huang SY, Hu YB, Li JC. Delayed postoperative radiotherapy might improve the long-term prognosis of locally advanced esophageal squamous cell carcinoma. Transl Oncol. 2021;14(1):100956. 33227662 10.1016/j.tranon.2020.100956 PMC7689552

[48] Tan Z, Ma G, Yang H, Zhang L, Rong T, Lin P. Can lymph node ratio replace pn categories in the tumor-node-metastasis classification system for esophageal cancer? J Thorac Oncol. 2014;9(8):1214–21. 25157776 10.1097/JTO.0000000000000216

[49] Shao Y, Geng Y, Gu W, Ning Z, Huang J, Pei H, Jiang J. Assessment of Lymph Node ratio to replace the pN categories system of classification of the TNM System in Esophageal squamous cell carcinoma. J Thorac Oncol. 2016;11(10):1774–84. 27393473 10.1016/j.jtho.2016.06.019

[50] Li X, Xu J, Zhu L, Yang S, Yu L, Lv W, Hu J. A novel nomogram with preferable capability in predicting the overall survival of patients after radical esophageal cancer resection based on accessible clinical indicators: a comparison with AJCC staging. Cancer Med. 2021;10(13):4228–39. 34128338 10.1002/cam4.3878 PMC8267131

[51] Duan J, Deng T, Ying G, Huang D, Zhang H, Zhou L, Bai M, Li H, Yang H, Qu Y, Wang X, Ba Y. Prognostic nomogram for previously untreated patients with esophageal squamous cell carcinoma after esophagectomy followed by adjuvant chemotherapy. Jpn J Clin Oncol. 2016;46(4):336–43. 26819278 10.1093/jjco/hyv206 PMC4886130

[52] Cao J, Yuan P, Wang L, Wang Y, Ma H, Yuan X, Lv W, Hu J. Clinical Nomogram for Predicting Survival of Esophageal Cancer patients after Esophagectomy. Sci Rep. 2016;6:26684. 27215834 10.1038/srep26684 PMC4877645

[53] Zheng B, Chen M, Chen C, Xiao J, Cai B, Zhang S, Liang M, Zeng T, Chen H, Wu W, Xu G, Zheng W, Zhu Y, Chen C. Adjuvant chemoradiotherapy for patients with pathologic node-positive esophageal cancer following radical resection is associated with improved survival. Ann Transl Med. 2020;8(24):1633. 33490145 10.21037/atm-20-4893 PMC7812226

[54] Yang W, Liu F, Xu R, Yang W, He Y, Liu Z, Zhou F, Heng F, Hou B, Zhang L, Chen L, Zhang F, Cai F, Xu H, Lin M, Liu M, Pan Y, Liu Y, Hu Z, Chen H, He Z, Ke Y. Is adjuvant therapy a better option for esophageal squamous cell carcinoma patients treated with esophagectomy? A prognosis prediction model based on multicenter real-world data. Ann Surg, 2021. 10.1097/SLA.0000000000004958 34091512

[55] Su D, Zhou X, Chen Q, Jiang Y, Yang X, Zheng W, Tao K, Wu J, Yan Z, Liu L, Wu S, Mao W. Prognostic Nomogram for thoracic esophageal squamous cell carcinoma after Radical Esophagectomy. PLoS ONE. 2015;10(4):e0124437. 25893524 10.1371/journal.pone.0124437 PMC4404051

[56] Zheng Y, Fu S, He T, Yan Q, Di W, Wang J. Predicting prognosis in resected esophageal squamous cell carcinoma using a clinical nomogram and recursive partitioning analysis. Eur J Surg Oncol. 2018;44(8):1199–204. 29784506 10.1016/j.ejso.2018.04.011

[57] Xiao W, Liang H, Zhang H, Jia R, Yang Y, Wang Y, Tang P, Yu Z. Ratio between negative and positive lymph nodes is a novel prognostic indicator for patients with esophageal cancer: a surveillance, epidemiology and end results database analysis. Thorac Cancer. 2020;11(12):3490–500. 33034409 10.1111/1759-7714.13688 PMC7705634

[58] Liu JS, Huang Y, Yang X, Feng JF. A nomogram to predict prognostic values of various inflammatory biomarkers in patients with esophageal squamous cell carcinoma. Am J Cancer Res. 2015;5(7):2180–9. 26328248 PMC4548329

[59] Zhu Y, Yao W, Xu BC, Lei YY, Guo QK, Liu LZ, Li HJ, Xu M, Yan J, Chang DD, Feng ST, Zhu ZH. Predicting response to immunotherapy plus chemotherapy in patients with esophageal squamous cell carcinoma using non-invasive radiomic biomarkers. BMC Cancer. 2021;21(1):1167. 34717582 10.1186/s12885-021-08899-x PMC8557514

[60] Cao K, Ma T, Ling X, Liu M, Jiang X, Ma K, Zhu J, Ma J. Development of immune gene pair-based signature predictive of prognosis and immunotherapy in esophageal cancer. Ann Transl Med. 2021;9(20):1591. 34790797 10.21037/atm-21-5217 PMC8576717

