# Supplementary material for: Survival risk stratification based on prognosis nomogram to identify patients with esophageal squamous cell carcinoma who may benefit from postoperative adjuvant therapy
Source: BMC Cancer. 2024 Oct 29;24:1330. doi: 10.1186/s12885-024-13085-w (PMC11520824; doi:10.1186/s12885-024-13085-w)
Supplement: Supplementary file 1 — Supplementary Material 1 [file 12885_2024_13085_MOESM1_ESM.pdf]

Dear Mr./Ms. _________:

I am entrusted by your attending physician, Dr. _________, to inquire about the current condition of Mr./Ms. _________. We sincerely hope to receive a truthful and effective response from you. We will strictly keep confidential all the information you provide.

If you are replying by mail, please use the enclosed envelope. If it is inconvenient for you to reply by mail, please call us at 025-68306093. Thank you for your cooperation!

Best regards,

Department of Radiation Oncology,

The First Affiliated Hospital of Nanjing Medical University

Please fill in or select the corresponding contents in the form below (check the box ☐).

Follow-up Records of Patients with Esophageal Cancer

Name: *Sex:* *Age:* *Inpatiet No.*

1. Current Eating Condition:
   ☐Normal Diet ☐Soft Food ☐Semiliquid Diet
   ☐Liquid Diet ☐Unable to Eat

2. Have you experienced acid reflux or heartburn after treatment?
   ☐Yes ☐No

3. Weight Change:
   ☐Increased ☐No Change ☐Decreased

4. Physical Condition:
   ☐Normal Work ☐Light Work
   ☐Self-Care ☐Unable to Self-Care

5. Have you visited a local hospital for follow-up?

☐No

☐Yes (Date: ____ Year ____ Month ____ Day)

Follow-up Results:

Blood Routine Test: ___________________________

Liver and Kidney Function: _____________________

Chest X-ray or CT Scan: ________________________

Esophageal Barium Meal: _______________________

Abdominal Ultrasound: _________________________

6. Have you received any treatment after surgery?
   ☐No
   ☐Yes
      ☐ Chemotherapy (Please specify the time and medication: ____________)
      ☐ Radiotherapy (Please specify the time, area, and number or dosage of radiation: _______________)

7. Please provide your phone number (landline or mobile): ____________________
   New Contact Address: __________________________________
   Postcode: ____

8. Others: (If deceased, please indicate the time: ____ Year ____ Month ____ Day; Cause of Death: Esophageal Cancer ☐ Other Cancer ☐ Other ☐)

Follower-up Respondent Signature: ___________________________
Date: _______________________
