# Supplementary material for: Survival risk stratification based on prognosis nomogram to identify patients with esophageal squamous cell carcinoma who may benefit from postoperative adjuvant therapy
Source: BMC Cancer. 2024 Oct 29;24:1330. doi: 10.1186/s12885-024-13085-w (PMC11520824; doi:10.1186/s12885-024-13085-w)
Supplement: Supplementary file 2 — Supplementary Material 2 [file 12885_2024_13085_MOESM2_ESM.docx]

| **Table S1. Baseline Clinicopathologic Characteristics of Selected Patients** | | | | | | | |
| --- | --- | --- | --- | --- | --- | --- | --- |
| Characteristics | All patients |  | Surgery |  | Surgery + Adjuvant |  | Surgery + chemotherapy |
|  | N=399(%) |  | n=270(%) |  | n=129(%) |  | n=103(%) |
| Age |  |  |  |  |  |  |  |
| ≤71 | 350(87.7) |  | 232(85.9) |  | 118(91.5) |  | 96(93.2) |
| > 71 | 49(12.3) |  | 38(14.1) |  | 11(8.5) |  | 7(6.8) |
| Sex |  |  |  |  |  |  |  |
| Male | 296(74.2) |  | 191(70.7) |  | 105(81.4) |  | 85(82.5) |
| Female | 103(25.8) |  | 79(29.3) |  | 24(18.6) |  | 18(17.5) |
| BMI |  |  |  |  |  |  |  |
| ＜18.5 | 29(7.3) |  | 21(7.8) |  | 8(6.2) |  | 6(5.8) |
| 18.5-24.9 | 283(70.9) |  | 190(70.4) |  | 93(72.1) |  | 74(71.8) |
| 25-29.9 | 82(20.6) |  | 56(20.7) |  | 26(20.2) |  | 21(20.4) |
| ≥30 | 5(1.3) |  | 3(1.1) |  | 2(1.6) |  | 2(1.9) |
| Having basic diseases |  |  |  |  |  |  |  |
| No | 227(56.9) |  | 152(56.3) |  | 75(58.1) |  | 57(55.3) |
| Yes | 172(43.1) |  | 118(43.7) |  | 54(41.9) |  | 46(44.7) |
| Smoking habit |  |  |  |  |  |  |  |
| No | 218(54.6) |  | 153(56.7) |  | 65(50.4) |  | 50(48.5) |
| Yes | 181(45.4) |  | 117(43.3) |  | 64(49.6) |  | 53(51.5) |
| Alcohol consumption habit |  |  |  |  |  |  |  |
| No | 272(68.2) |  | 194(71.9) |  | 78(60.5) |  | 58(56.3) |
| Yes | 127(31.8) |  | 76(28.1) |  | 51(39.5) |  | 45(43.7) |
| Family history |  |  |  |  |  |  |  |
| No | 353(88.5) |  | 233(86.3) |  | 120(93.0) |  | 94(91.3) |
| Yes | 46(11.5) |  | 37(13.7) |  | 9(7.0) |  | 9(8.7) |
| EOCG |  |  |  |  |  |  |  |
| 0 | 117(29.3) |  | 105(38.9) |  | 12(9.3) |  | 12(11.7) |
| 1 | 244(61.2) |  | 142(52.6) |  | 102(79.1) |  | 78(75.7) |
| 2 | 38(9.5) |  | 23(8.5) |  | 15(11.6) |  | 13(12.6) |
| Tumor location |  |  |  |  |  |  |  |
| Upper | 20(5.0) |  | 12(4.4) |  | 8(6.2) |  | 6(5.8) |
| Middle | 176(44.1) |  | 117(43.3) |  | 59(45.7) |  | 46(44.7) |
| Lower | 203(50.9) |  | 141(52.2) |  | 62(48.1) |  | 51(49.5) |
| Tumor length(cm) |  |  |  |  |  |  |  |
| ＜3 | 191(47.9) |  | 150(55.6) |  | 41(31.8) |  | 29(28.2) |
| ≥3 | 208(52.1) |  | 120(44.4) |  | 88(68.2) |  | 74(71.8) |
| Differentiation Grade |  |  |  |  |  |  |  |
| Well | 25(6.3) |  | 23(8.5) |  | 2(1.6) |  | 1(1.0) |
| Moderately | 186(46.6) |  | 136(50.4) |  | 50(38.8) |  | 40(38.8) |
| Poorly | 188(47.1) |  | 111(41.1) |  | 77(59.7) |  | 62(60.2) |
| LVSI |  |  |  |  |  |  |  |
| No | 345(86.5) |  | 246(91.1) |  | 99(76.7) |  | 78(75.7) |
| Yes | 54(13.5) |  | 24(8.9) |  | 30(23.3) |  | 25(24.3) |
| Perineuronal invasion |  |  |  |  |  |  |  |
| No | 350(87.7) |  | 245(90.7) |  | 105(81.4) |  | 84(81.6) |
| Yes | 49(12.3) |  | 25(9.3) |  | 24(18.6) |  | 19(18.4) |
| LNR(%) |  |  |  |  |  |  |  |
| ＜12 | 338(84.7) |  | 245(90.7) |  | 93(72.1) |  | 71(68.9) |
| ≥12 | 61(15.3) |  | 25(9.3) |  | 36(27.9) |  | 32(31.1) |
| Surgery approach |  |  |  |  |  |  |  |
| Sweet | 30(7.5) |  | 20(7.4) |  | 10(7.8) |  | 6(5.8) |
| Ivor Lewis | 302(75.7) |  | 202(74.8) |  | 100(77.5) |  | 85(82.5) |
| McKeown | 67(16.8) |  | 48(17.8) |  | 19(14.7) |  | 12(11.7) |
| pT stage |  |  |  |  |  |  |  |
| 1 | 138(34.6) |  | 125(46.3) |  | 13(10.1) |  | 11(10.7) |
| 2 | 79(19.8) |  | 57(21.1) |  | 22(17.1) |  | 18(17.5) |
| 3 | 182(45.6) |  | 88(32.6) |  | 94(72.9) |  | 74(71.8) |
| pN stage |  |  |  |  |  |  |  |
| 0 | 234(58.6) |  | 205(75.9) |  | 29(22.5) |  | 15(14.6) |
| 1 | 135(33.8) |  | 55(20.4) |  | 80(62.0) |  | 69(67) |
| 2 | 30(7.5) |  | 10(3.7) |  | 20(15.5) |  | 19(18.4) |
| pTNM stage |  |  |  |  |  |  |  |
| I | 116(29.1) |  | 116(43.0) |  | 0(0) |  | 0(0) |
| II | 141(35.3) |  | 99(36.7) |  | 42(32.6) |  | 26(25.2) |
| III | 142(35.6) |  | 55(20.4) |  | 87(67.4) |  | 77(74.8) |
|  |  |  |  |  |  |  |  |
